# The Influence of Fly Ash on the Mechanical Properties of Water Immersed All Waste Composites

**DOI:** 10.3390/polym14101957

**Published:** 2022-05-11

**Authors:** Mihaela Cosnita, Monica Balas, Cristina Cazan

**Affiliations:** Renewable Energy Systems and Recycling Research Center, Transilvania University of Brasov, 500036 Brasov, Romania; monica_balas@unitbv.ro

**Keywords:** fly ash, end of life tire rubber, rubber-PET-HDPE-wood composites, wood waste, mechanical properties

## Abstract

The paper presents new value-added composite materials prepared by recycling tire rubber, polyethene terephthalate (PET), high-density polyethene (HDPE), wood sawdust, and fly ash. The composites were manufactured through the compression molding technique for three temperatures (150 °C, 160 °C, and 190 °C) previously optimized. The addition of fly ash as reinforcement in polymer blends is a viable route to improve the composite” properties. The paper aims to assess the effect of fly ash on the mechanical properties and water stability of the new all waste composites considering their applications as outdoor products. The static tensile (stress-strain behavior) and compression properties of the composites were tested. The fly ash composites were characterized in terms of wetting behavior and surface energies (contact angle measurements); chemical structure of the new interface developed between composite” components (FTIR analysis), crystalline structure (XRD analysis), surface morphology and topography (SEM, AFM). The addition of fly ash promoted the development of the hybrid interfaces in the new composites, as FTIR analysis has shown, which, in turn, greatly improved the mechanical and water resistance. The novel all waste composites exhibited lower surface energies, larger contact angles, and smoother morphologies when compared to those with no fly ash. Overall, the study results have revealed that fly ash has improved the mechanical strength and water stability of the composites through the formation of strong hybrid interfaces. The study results show optimal water stability and tensile strength for 0.5% fly ash composites cured at 190 °C and optimal compressive strength with good water stability for 1% fly ash composite cured at 150 °C.

## 1. Introduction

Nowadays, several worldwide issues derive from the huge amounts of waste disposal, which seriously harms the environment and humanity’s health. The reuse of and waste recycling could save 600 billion euros for EU companies. Consequently, gas emissions could be reduced by up to 4% per year [1]. Tire rubber wastes represent an important waste category with a continuous growth rate and negative impact on the environment. Globally, about 1.5 billion tires are discarded per year in addition to the already accumulated large volume [2]. The management of waste tire rubbers is often reduced only at energy recovery by their incineration in a cement kiln, paper plants, etc. [3], but there is a huge loss of materials at the same time that causes a negative impact on the environment. The most sustainable method for recycling rubber tires could be by grinding and blending them with other polymers in order to achieve new polymer composites [4].

On the other hand, plastic-based products are a part of our daily life because of their low cost, density, high strength, and their resistance to corrosion and weathering. Worldwide, the production of plastics doubled from 2000 to 2020, reaching 360 million tons. There are many single-use plastic products (PET, HDPE, PS, PP), and after use, they are thrown away, thus increasing the environmental burden and causing detrimental effects on the quality of water, soil, and human healthy consequently [5,6]. 

Government legislative efforts are conducted to recycle end-of-life plastic-based products into value-added products [7].

However, reports on the recycling of plastics in a sustainable way are scarce. Often, the plastic products coming from beverages and the food industry are thrown everywhere, and finally, they reach the sea and endanger marine life.

The use of biomass fillers such as wood, sisal, flax, and so on, to produce composite materials offers a series of advantages, which include a low density, less processing equipment damage, flexibility, biodegradability, and minimal health hazards [8,9].

Wood sawdust, resulting from the exploitation of wood, could harm the environment when there is improper disposal. The strength of wood is 20 times higher than of the HDPE being used as reinforcement in plastic composites. The advantages of using wood in WPC over classic fibers are related to its high availability, low weight and cost, thermal and insulation properties, and a lack of abrasiveness to equipment [10].

There are reports on the development of wood-plastic composites (WPC) and rubber-wood-plastic composites (RubWPC), which have a low density and good mechanical properties [11,12,13,14,15,16]. The mechanical properties of WPC are determined by the interface adhesion between the wood and the polymer matrix. There are many reported methods for the improvement of polymer-wood compatibilities, such as grafting the maleic anhydride to a polymer, adding coupling agents, treating the wood through mercerizing, plasma, corona, and so on [17,18,19,20]. These wood treatments promote the wood–polymer interface adhesion, but they are difficult to be applied on a large scale due to their cost and because they often use toxic chemical compounds.

However, sustainable and economical methods could be applied for the recycling of most discarded waste types (rubber, plastic, wood, etc.) by manufacturing performant and water stability RubWPC through the addition of inorganic fillers.

Moreover, the addition of inorganic fillers in the form of waste into a polymer matrix leads to the formation of the hybrid interfaces, which, in turn, greatly improve the mechanical, physical, thermal, flame retardant, water stability, and durability properties of organic–inorganic (hybrid) composites [21,22,23,24].

Silica is often used for improving the wood–polymer interface in order to achieve new value-added products by increasing the synergistic effects of both components’ properties [25].

The available FA, the waste generated from heating a power plant (enriched with silica), could be an important candidate for use as a filler in rubber-plastic-wood composites.

Fly ash is widely used as a supplementary cementitious material in concrete manufacturing. It improves the workability and durability of concrete while reducing the costs of concrete products [26,27,28].

To the best of our knowledge, there are no reports on the influence of FA on water stability and the mechanical properties of all waste composites manufactured from waste rubber, PET, HDPE, and wood.

The new hybrid waste composites, including organic and inorganic fillers in the rubber matrix, could reduce the environmental burden while also providing increased performance products for outdoor applications, such as paving slabs, covers for play and sports grounds, highway walls, and so on.

The paper aims to assess the effect of fly ash on the mechanical properties and water stability of the new all waste composites developed by recycling together most discarded wastes such as tire rubber, PET, HDPE, and wood.

## 2. Experimental Methodology

### 2.1. Materials

The materials used in this study were waste tire rubber from Granutech Recycling (Suceava, RO, USA), PET flakes from used bottles, small amounts of high-density polyethene flakes (HDPE, PET from Teli Company), wood sawdust (fir 1–2%, and beech 98–99%) from Wood Engineering Department, Transilvania University of Brasov, Romania, with a natural moisture content of 5.28% and fly ash (FA) coming from the Heating Plant CET Brasov, RO. FA is a cheap material and is a poly-oxide compound with a high weight percentage of SiO_2_ (53.32 wt%), Al_2_O_3_ (22.05 wt%), and Fe_2_O_3_ (8.97 wt%). As per ASTM standard C-618-2a [29], this FA is of F type (the SiO_2_, Al_2_O_3_, and Fe_2_O_3_ content exceed 70%) [30]. All of the materials were milled to 1 mm-sized particles.

### 2.2. Methods

All waste rubber-PET-HDPE-wood composites were prepared through the compression molding method using a thermostat oven (type ECv 200–300) for thermal curing. The thermal processing duration was one hour at 150 ± 5 °C (samples 1S type), 160 ± 5 °C (samples 2S type), 190 ± 5 °C (samples 3S type) temperatures optimized in our previous work [31]. The composite components mass ratio previously optimized and the codes of the samples without fly ash (FA) are:

- rubber:PET:HDPE:wood = 80:10:5:5, samples 1S (cured at 150 °C), 2S (cured at 160 °C) and 3S (cured at 190 °C);

The mass ratio of the fly ash composite components is rubber:PET:HDPE:wood:FA = (80 − x):10:5:5:x, with the fly ash weight percentage ranging from 0.5 to 2 wt% with a 0.5 wt% step. The fly ash added composites are denoted as follows in Table 1.

Two set samples of each series were prepared: the S series (without FA) and the S-FA series (with FA). The two sets of each series of samples without FA and with FA (denoted as S and S_FA, respectively) were thermally processed at the previously optimized temperatures of 150 °C, 160 °C, and 190 °C [31]. A sample set of each series (S and S-FA, respectively) were kept for three days in laboratory condition, and those from the second set of each composite series were immersed for 5 days in tap water (Total Hardness = 14.5 dGH) and then were dried in an open air laboratory before their characterization. Five representative samples of each series were mechanically tested.

### 2.3. Characterization Techniques

**Mechanical tests:** Tensile strength (*σ_t_*) and Young modulus (E) were evaluated with Z020, Zwick/Roell equipment (DE) at a traction speed of 100 mm/min. The compression resistance (R_C_) was tested on the same mechanical testing equipment, according to SR EN ISO 527-4:2000.

**Scanning electron microscopy (SEM)** and **energy-dispersive X-ray spectroscopy** **(EDX) analysis:** Micrographs were obtained by using a scanning electron microscope (SEM, JP), Hitachi, S3400N, type II, and quantitative elemental analysis of the samples was performed with EDX (Thermo, Ultra Dry, Noran System 7, NSS Model, 2000000counts/sec) and the sensitivity down to a few atomic percent. 

**Atomic force microscopy (AFM) analysis:** The surface morphology and topography of the nanocomposite were investigated by atomic force microscopy (AFM NT-MDT model NTEGRA PRIMA EC). The images were taken in semi-contact mode with a “GOLDEN” silicon cantilever (NCSG10, force constant 0.15 N/m, tip radius 10 nm).

**Fourier Transforms Infrared Spectroscopy (FTIR) analysis:** was performed using a Spectrum Bruker spectrophotometer (KR); the spectra were recorded in reflectance mode, in the 500 to 4500 cm^−1^ range, after 16 scans, with a resolution of 4 cm^−1^.

**X-ray diffraction (XRD):** The crystallinity data were collected over the range 2θ = 10 ~ 60° in the fixed time mode, with a step interval of 0.01°, at 25°, using a Bruker Advanced D8 diffractometer (CuK_α1_ radiation, with 1.5406 Å wavelength, at 40 kV, 20 mA).

**Contact angle measurements:** The measurements of the composite surfaces were performed with an OCA-20 Contact Angle System (Data Physics Instruments), based on the sessile drop method. The surface energies were calculated using the Owens, Wendt, Rabel, and Kaelble (OWRK) method [32]. The two liquids used in the experiments were: water (σ_H2O_ = 72.10 mN/m, σ^p^_H2O_ = 52.20 mN/m and σ^d^_H2O_ = 19.90 mN/m) and glycerol (σ_glycerol_ = 73.40 mN/m, σ^p^_glycerol_ = 37.00 mN/m and σ^d^_glycerol_ = 36.40 mN/m) and the total surface energy, polar, and the dispersive components were obtained using Equation (1) [32]:

(1) $$\sigma_{SL}=\sigma_{S}+\sigma_{L}-2\left(\sqrt{\sigma_{S}^{d}\cdot \sigma_{L}^{d}}+\sqrt{\sigma_{S}^{p}\cdot \sigma_{L}^{p}}\right)$$

$\sigma_{SV}$—surface energy; *σ_S_*—surface tension of testing liquid; *σ_L_*—surface tension of solid surface; $\sigma_{SV}^{d}$, $\sigma_{SV}^{p}$—dispersive and polar component of the surface energy.

## 3. Results and Discussions

### Mechanical Tests

Composite materials should meet the required mechanical properties, such as tensile, compressive, and impact strength depending on the targeted applications. The mechanical behavior of the composite materials is strongly influenced by interfacial zone strength. Interfacial adhesion plays a key role that greatly entails the composite’s mechanical, thermal, and durability properties. The interfacial adhesion in composite systems could be tailored mainly by three mechanisms, mechanical, mechanical-chemical, or chemical bonds between the components of the composite system. The nature of the interfacial adhesion in a composite system is strongly dictated by the technological and compositional parameters, filler dispersion degree, filler properties, such as wetting, shape and size, components properties, and so on. 

A small amount of flay ash (up to 2%) was added in order to avoid fly ash cluster formation as the filler particle interactions are larger than the interaction of the composite components in the interfacial zone. By adding a small amount of fly ash, cluster formation is avoided. The interfacial linkage between the fly ash and polymer matrix is crucial to the fly ash composite’s mechanical strength [33,34,35]. 

The assessment of the composite behavior under wet conditions represents a prerequisite when outdoor applications are being pursued. That is why the study evaluated the mechanical strength before and after water immersion of the novel hybrid composites. 

The previously optimized temperatures valorize the mechanical properties and benefit of the wood incorporated into the rubber-PET-HDPE blend. Wood composites obtained at higher curing temperatures lead to the decomposition of the wood components, thus reducing the binding effect between composite components. Crystalline cellulose from the wood greatly enhances the mechanical properties of the rubber—plastic-based composites when the composite is cured at temperatures below 200 °C [36]. These two series of composites were mechanically tested, and the results are presented in Table 2. The addition of wood sawdust significantly influences the mechanical properties of the rubber–plastic composites.

**Figure 1:**
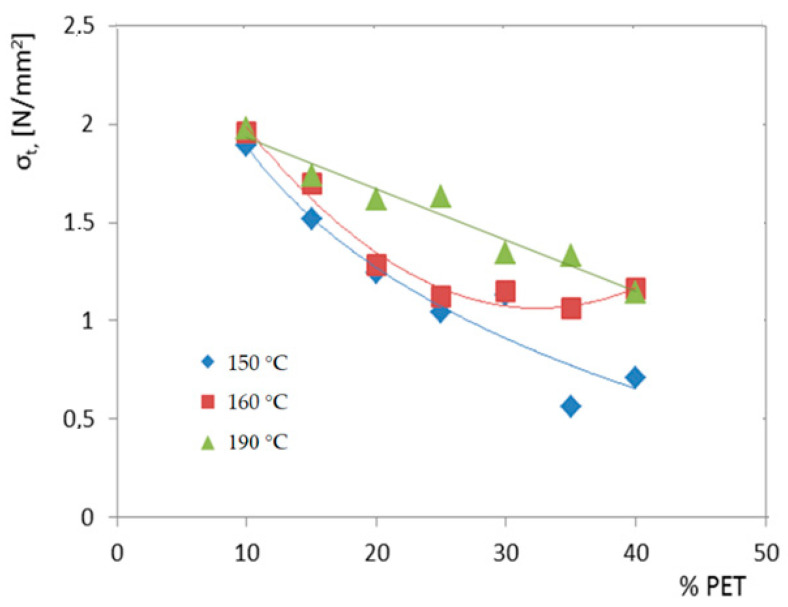
Variation of tensile strength with % PET and processing temperature.

Up to 40 wt% of the PET was incorporated in the rubber matrix for the three optimized temperatures, as is shown in Figure 1. 

The tensile strength of the all-waste composite drastically decreased when the PET content was increased due to the weakening interface with PET particle agglomerations, Figure 1. The agglomeration of PET at the interface zone hampers the stress transfer from the matrix to filler, as tensile strength tests have revealed. The highest tensile strength was recorded to be 10 wt% for the PET composites. That is why in developing novel composites with rubber, HDPE, wood, and fly ash 10% wt PET was considered.

Five representative samples (with fly ash) of each series (before and after water immersion) were mechanically tested, and the average values are summarized in Table 2.

**Figure 2:**
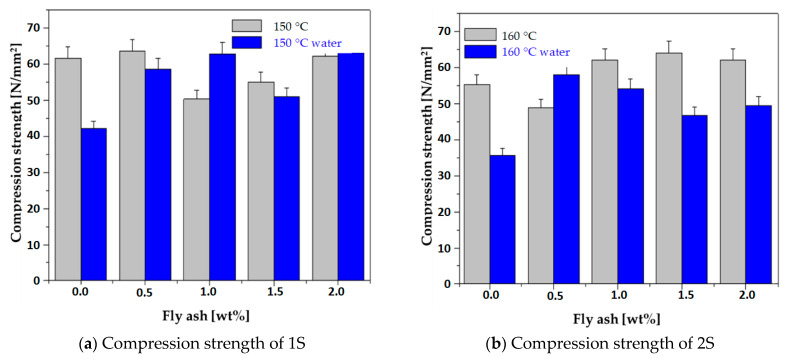

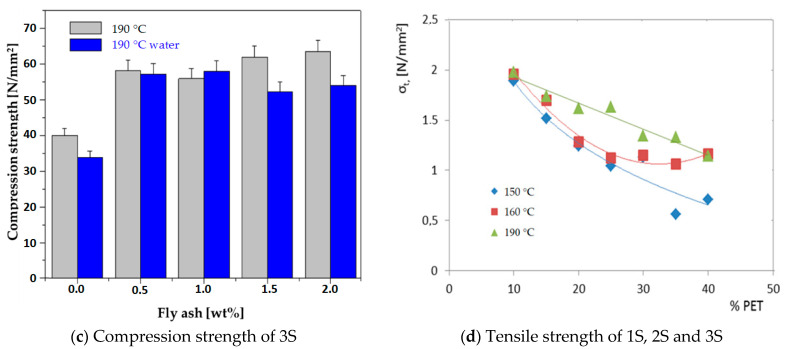
Mechanical properties of all waste composites before and after water immersion.

By comparing the pristine composite series cured at 150 °C, 160 °C, and 190 °C, the tensile strength increased as the curing temperature increased, and we recorded a decreasing trend with the increased amount of FA incorporated into the rubber-plastic-wood blend. Therefore the lowest tensile strength corresponds to 2% in the FA highest sample obtained at 150 °C (1S_FA—4), and the highest was recorded for the 0.5% FA composite cured at 190 °C (3S_FA—1), as is shown in Figure 2.

The mechanical properties of a composite system’s tensile strength best reflect the interface strength. The curing temperature strongly impacts the composite’s mechanical strength. The rubber matrix and HDPE undergo a viscous to fluid phase at the curing temperatures of 150 °C and 160 °C, thus supporting the development of physical–chemical interfaces. Instead, the hydrophilic hemicellulose wood component starts to degrade, and interfaces between rubber, FA, wood, and HDPE are more likely to be formed [31].

The composite cured at 190 °C registered the best tensile strength, and the behavior could be due to the increase in the interface’s density. At this temperature, PET undergoes a viscous fluid thermal transition and supports the interface zone extension. The processing temperature of 190 °C compared to the lower ones ensures a tight contact between the composite’s components, thus favoring the widening of the interface’s zone. The thermal transitions of the rubber, HDPE, and PET alongside the increased amount of hydrophilic wood components degraded favor the development of rubber-PET/HDPE-wood/-rubber-HDPE/rubber-FA interfaces following both mechanisms of physical–chemical bonding and mechanical sticking. The composite’s interface is further investigated in the FTIR analysis section.

In the 150 °C and 190 °C composites, we recorded a slight increase in tensile strength, Figure 2. The absorption/desorption mechanism of water molecules that enter the capillary structure, then the micro-cracks, and finally the water diffusion of the composite explain this behavior. The 1S_FA—2 exhibits good stability in mechanical strength even after water immersion. The water stability of 1S_FA—2 is entailed by the particular behavior of water molecules, which act as a plasticizing agent when they enter the composite’s capillarity structure. The plasticizing water alongside the wood components, which act as a binder in the composite with waste rubber and HDPE [31], enhances the interface strength between the composite’s components. 

The compressive strength of the pristine series registered an increasing trend as a small fly ash amount was added to the rubber-PET-HDPE-wood blend, Figure 2. The highest compressive strength was assigned to the 2% fly ash composites for all three optimized temperatures. This mechanical behavior is owed to the mechanical strength of fly ash, its rigidity, and especially due to its high SiO_2_ content, which exhibits a high affinity for the rubber matrix and thus extending the contact area. There are reports on fly ash’s high affinity for rubber matrices [37,38]; thus, hybrid rubber–fly ash interface development is likely to be developed. It is worthy to note that the 160 °C sample recorded the best compression increase after the addition of FA. This behavior was noticed in a previous study for the composite series treated at 160 °C [31]. The rubber-plastic-wood composite produced at T = 160 °C exhibits a particular compression strength increase when inorganic fillers are added. The explanation is related to the composite densification when FA particles enter the sponge-like structure of the rubber-plastic-wood blend, reducing the macromolecular chains’ mobility and highly contributing to the increase in the composite’s rigidity, as seen in Figure 2. The 190 °C composites with no FA recorded over a 50% lower compression strength compared to those cured at 150 ^0^ C and 160 °C, as seen in Figure 2. The decrease in mechanical strength is directly linked to the partial degradation of the wood with a corresponding loss in the composite’s mechanical strength. The 3S_FA—4 registered an outstanding increase in compression strength, with approximately 63 MPa when FA was inserted into the rubber-plastic-wood blend. The composite’s resistance increase when fly ash was added could be explained by the rigid and rich silica FA cenospheres, which enter into the composite’s capillaries and improve the composite’s density.

The water immersion of fly ash composites led to a slight decrease in the compressive strength, as seen in Figure 2. The water immersion of the composites could induce a possible flexibilization of their structure with a subsequent loosening in the structure. However, the samples with 1% fly ash prepared at 150 °C, denoted as 1S_FA—2, and the 0.5% fly ash sample prepared at 160 °C (2S_FA—1) registered a significant compression strength increase after their water immersion, 24.75% and 18.96%, respectively. These results prove the synergistic effect of wood, water (plasticizing), and the proper amount of FA on the strength increase in the composite’s interface. The FA’s cenospheric structure, as was in the SEM analysis section, enters the capillary structure of the composite rubber matrix and leads to a high potential for rubber–FA crosslinking. 

The bound water effect (possible with the inorganic filler modifications) is reflected in the tensile strength of composites registered after their immersion in water. The tensile strength of the water immersed composites recorded a slow increase rate, with the highest tensile strength for the 0.5% FA composite prepared at 190 °C, as seen in Figure 2. 

These composites could be promising for use in outdoor products (paving slabs, railways, coverings for playgrounds, and sports fields), considering that the matrix can encapsulate the wood components and improve their resistance to humidity and prevent swelling. 

**Surface energy measurements**

Considering the outdoor applications of the developed composite based on organic and inorganic phases, the wetting behavior is of the utmost importance because of its influence on aging. The surface energies of the fly ash composites compared to those without FA were determined based on contact angle measurements. The surface energies of composites are strongly influenced by the surface chemical structure, morphology, surface roughness, the tested liquid type, and the interaction between liquid–surface molecules. The hydrophobic nature of polymer materials with their poor polarity leads to a low contact angle value. The FA composites to be used in outdoor products are required to be resistant to wet environments in order to preserve their interface strength and mechanical characteristics, respectively. The contact angles of the composite surface with both liquid water and glycerol and surface energies $\sigma_{SV}$ with their components dispersive and polar ($\sigma_{SV}^{d}$$\sigma_{SV}^{p}$) before and after water immersion are summarized in Table 3.

Almost all of the fly ash composites exhibit low surface energies for all three optimized temperatures. The composites denoted as 1S_FA—3 and 3S_FA—3 present a slight surface energy increase compared to all of the others, but it is worthy to note that their dispersive component is prevalent, as seen in Table 3. The contact angle measurement results well match the mechanical tests, which registered good compression strength, thus proving the stiffening effect of FA related to its high content of oxides. The high contact angle values and the low value of the polar components support the FA composite’s hydrophobicity; thus, it has a high potential for application in outdoor products in wet environments. The surface energies and contact angles (with water and glycerol) of water immersed FA composites have shown a similar trend as the unimmersed ones, thus outlining the hydrophobic character of FA composites. 

Therefore, poor polarity and low surface energy FA composites well match the requirements of outdoor products (e.g. building materials, tiles, covers for several grounds, pillar sleeves for a carpark and so on).

**FTIR analysis**

The possible hybrid interfaces in the novel all-waste composites were investigated by Fourier Transformed Infrared (FTIR) spectroscopy. During the thermal processing of the polymeric blend with the fly ash, as a result of oxidative processes or reciprocal affinity alongside mechanical adhesion, physical, and/or chemical bonds could be established between the composite’s components.

**Figure 3:**
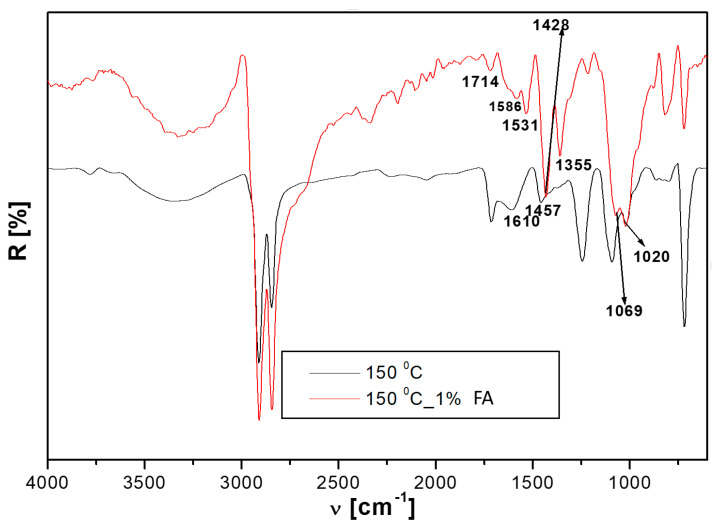
FTIR spectra of samples cured at 150 °C with and without FA, coded as 1S_FA—2 and 1S.

The FTIR bands of the FA composite with the best combination of mechanical properties (1S_FA—2) were investigated and compared to that without FA, as shown in Table 4. The FA addition to the rubber-plastic-wood blend brought significant changes to the FTIR spectrum, as can be seen in Figure 3:

- the 1610 cm^−1^ band assigned to C=C (rubber) or deformation vibration of water from wood disappeared and instead appeared as two bands, 1586 and 1531 cm^−1^. The first corresponds to the lignin from wood and the second to the carboxylate group (–COO^−^), [32] or C=C from rubber. These changes could be explained by the hybrid interface formation through chemical bonding between rubber, wood, and FA;
- the shift of the of 1458 cm^−1^ (C=C from rubber) and 1245 cm^−1^ (C–O–C of PET or wood) to lower wavenumbers 1428 and 1215 cm^−1^, respectively; a new band at 1355 cm^−1^ between the previous occurred and was assigned to wood constituents (–CH_3_ from lignin/hemicellulose/polysaccharide –OH). These results indicate possible chemical interactions between rubber, PET, HDPE, wood, and FA compounds. The shifts of these bands toward lower wavenumbers could explain the interface’s flexibilization.
- the appearance of new bands from 1115 (of high intensity) and 537 cm^−1^ were assigned to silica and other oxides from FA, as seen in Figure 3. These new bands’ appearance corresponds to the possible chemical interactions between the matrix and the prevalent silica compounds from fly ash. These results support the mechanical test results, which indicated an increase in the mechanical strength of the composite with fly ash compared to those without fly ash.

**Figure 4:**
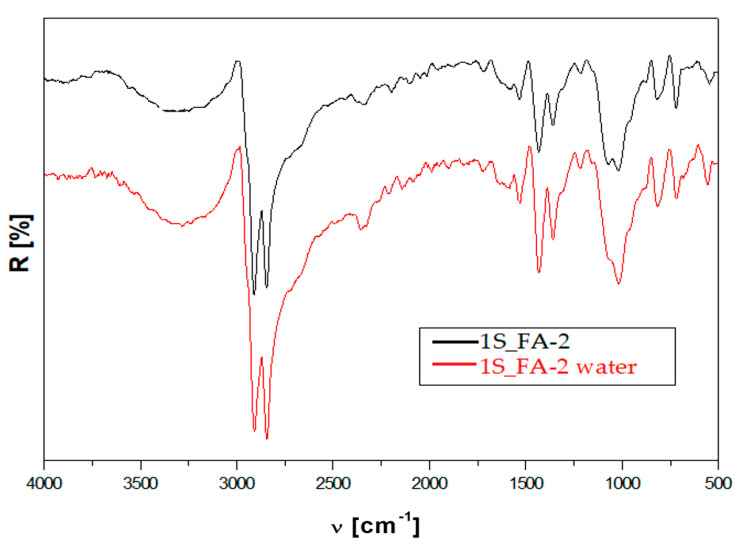
FTIR spectra of 1S_FA—2 before and after water immersion.

By comparing the FTIR spectra of the 1% FA composite, Figure 4, cured at 150 °C before and after immersion in water, the following changes were noticed, as seen in Table 5.

- The shift to higher wave numbers of 2842, 1722, 1369, 1219, and 719 cm^−1^ bands. These changes clearly indicate the increased rigidity of the composite structure, as explained by the plasticizing effect of the water in the composite capillaries. The FTIR results confirmed the mechanical test results, which registered an increase in the compression strength and stable tensile strength to water in the 1S_FA—2 sample.

The water immersion of the composite with FA led to some changes; 1069, 1020, and 874 cm^−1^ bands occurred as a single broader band at 1019 cm^−1^. This could be explained by the physical and chemical interactions between PET, wood, and FA made possible through hydrogen bonds. Furthermore, these interactions extend the interface zone, which, in turn, leads to an increase in mechanical strength, as was already noticed in the mechanical test results section.

**XRD analysis**

An XRD investigation was performed to identify the crystalline and amorphous domains of all waste composites with FA and their influence on the mechanical properties. The sample investigated 1S_FA—2 to explore the best combination of mechanical properties even under humidity and from an economic aspect, being manufactured at the lowest temperature (150 °C). The XRD results are presented compared to samples with no FA addition.

The crystallinity of this composite type is determined by the wood components with their crystalline cellulose and their nucleating agent role on the one hand and on the other hand because of inorganic filler [39,40,41,42]. There are reports on the PET nucleating role in wood and HDPE-based composites [43].

The higher crystallinity of the FA composite compared to 1S composites is explained by the increase in the ordered degree as a consequence of the rearrangement of the polymer macromolecular chains. The fly ash, due to its cenospheric shape and high affinity to the matrix, ensures tight contact over a large area with the rubber macromolecular chains. Consequently, the mobility of the macromolecular chains is diminished, which in turn leads to an increase in the ordered degree and a higher crystalline percentage, as shown in the XRD results.

Diffractograms of this composite type showed a broad peak due to the amorphous polymer–rubber matrix. The highest intensity peak is assigned to HDPE, with its high symmetry of macromolecules has the largest percentage of crystalline phase. 

**Figure 5:**
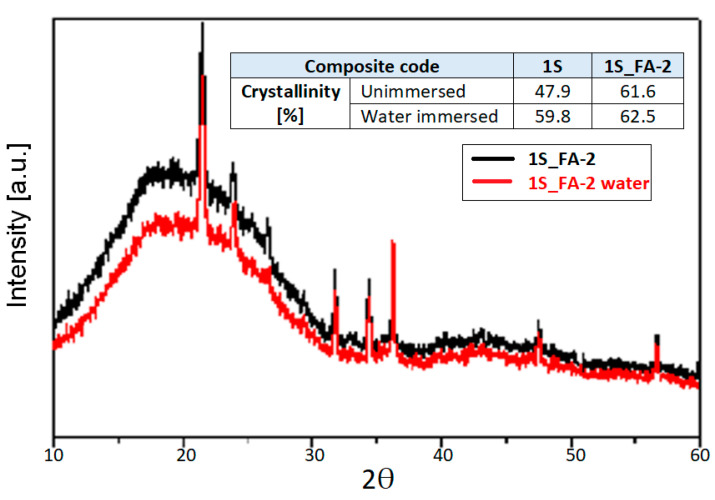
XRD diffractograms of 1S_FA—2 unimmersed and after water immersion.

A slight increase in the crystalline degree was noticed for the water-immersed 1S_FA—2, as can be seen in Figure 5. This result could be explained by the rearrangement of the macromolecular chains as the water molecules enter the composite capillarity structure. 

The crystalline degree of both unimmersed and water immersed 1S_FA—2 recorded similar values. These XRD results clearly confirm the strong hybrid interface between the components of the composite. These results match the FTIR and mechanical test results, which revealed for 1S_FA—2 the formation of new chemical bonds and good stability in the mechanical strength even after water immersion. 

One may conclude that the crystalline degree could support the composite resistance to water action, conferring thus a rigid matrix that is difficult to weaken or modify.

**SEM and AFM microscopy**

Scanning electron microscopy and atomic force microscopy were performed in order to investigate the quality of the surface morphology and interface structure. Therefore, a low-rugosity surface shows good linking between the composite components and a good interface strength. AFM images were taken over a 50 × 50 μm^2^ surface of the composites with the best combination of mechanical properties (1S_FA—2) compared to that with no FA (1S).

**Figure 6:**
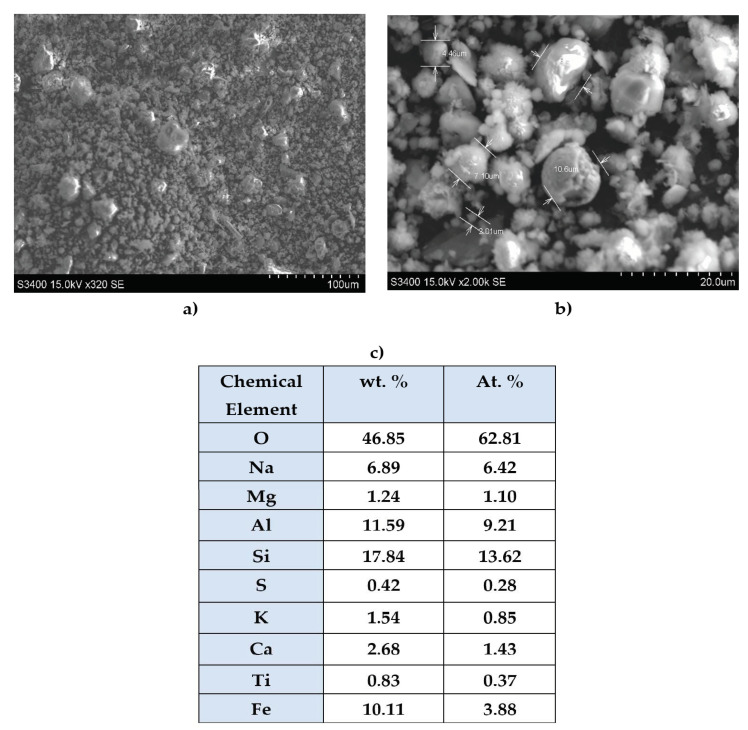
SEM morphology of fly ash (**a**,**b**) with its chemical elemental composition (**c**).

The SEM morphology images obtained by the secondary electrons of fly ash evidenced their cenosphere structure with a high specific surface, Figure 6. The FA cenospheres with a 50-times smaller diameter size than the rubber matrix are effective in filling the capillary structure of the rubber-plastic-wood blend, thus enhancing their density. The EDS analysis results pointed out the poly oxides-rich composition of FA with prevalent silica share. These results are in good agreement with the mechanical tests and FTIR results. The first composite strength increase through the composite structure densification due to the addition of FA cenospheres was recorded, and the FTIR results revealed the possible formation of the hybrid interface of rubber-FA type was due to the high silica affinity to the rubber matrix. 

**Figure 7:**
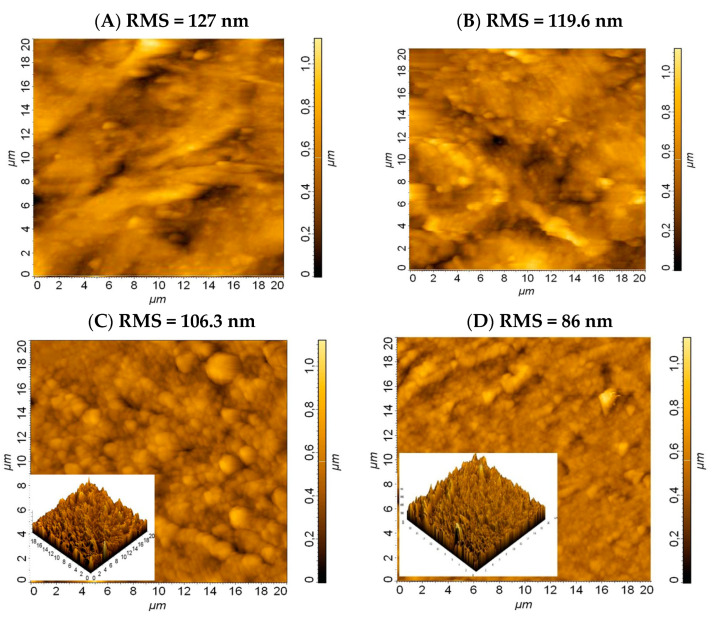
AFM images of 1S_FA—2 before and after water immersion of: (**A**) 1S; (**B**) 1S water immersed; (**C**) 1S_FA—2; (**D**) 1S_FA—2.

The AFM results revealed a decrease in rugosity (RMS = 106.3 nm) for the pristine FA composite (1S_FA—2) compared to the composite without FA (1S. RMS = 127 nm), Figure 7A,C. The FA composite with the fly ash cenospheres filling the capillarity structure of the composite led to a more compact and homogenous structure for 1S_FA—2 compared to 1S, as can be seen in Figure 7A,C. 

The smoother surface morphology of the FA composite compared to those with no FA may be explained by the good distribution of fly ash cenosphere in the matrix. This behavior is owed to the high affinity between the silica particles and the polymer matrix (rubber) confirmed by the low roughness values, as can be seen in Figure 7C, compared to the composite without FA in Figure 7A. Water-immersed FA composite presents a significant roughness decrease, 20% approximately, compared to that before immersion, Figure 7C,D. The water molecules that enter the capillary structure of the composite act as a plasticizing agent, smoothing the surface of the water-immersed 1S_FA—2 compared to that of the unimmersed, as can be observed from Figure 7C,D. The increase in surface smoothness of the water-immersed 1S_F A-2 is explained by the plasticizing effect of water, as can be clearly seen from their topography in the insets of Figure 7C,D.

The decrease in rugosity in the water-immersed 1S_FA—2 compared to the unimmersed one supports the XRD results, which recorded an increase in the degree of crystalization, and the mechanical tests, which registered an enhancement in the compressive strength.

## 4. Conclusions

The influence of fly ash (FA) cenospheres on the mechanical properties and water stability of the new all-waste composites based on tire rubber, PET, HDPE, and wood sawdust was assessed considering their applications as outdoor products. 

The synergistic effect of high stiffness and tensile strength wood alongside rich silica FA with its rigidity was reflected in the superior mechanical properties of the all-waste composites (S-FA type) compared to that without FA (S type). The high FA affinity to the rubber matrix and water as the plasticizer supports strong hybrid interface formation and thus the mechanical strength of rubber-PET-HDPE-wood-FA composites, even in wet conditions. The mechanical performance is well supported by FTIR analyses which outlined hybrid interface formation through chemical bonding. Optimal processing temperature and FA weight ratio in the rubber-PET-HDPE-wood blend are key factors in designing composites with stable mechanical features, even in wet environments. 

The best combination of mechanical properties was recorded for 1% FA samples cured at 150 °C and 0.5% FA samples cured at 190 °C. 

The water immersion of the rubber-PET-HDPE-wood-FA composite led to a high ordered degree, as the XRD results have shown. The water molecules act as a plasticizer smoothening the composite’s surface, as the AFM and contact angle measurements were confirmed by the low rugosity (RMS) and low surface energies, respectively.

The optimal mechanical strength and water stability correspond to the composite cured at 150 °C with 1% fly ash, which could be recommended for outdoor products such as paving slabs, covering playgrounds, and so on.

## Figures and Tables

**Table 1:** Codes of fly ash composites.

Samples Composition [wt%]	T [°C]	FA [%]	Samples Code
rubber:PET:HDPE:wood: FA = (80 − x):10:5:5:x	150	0.5	1S_FA—1
		1	1S_FA—2
		1.5	1S_FA—3
		2	1S_FA—4
	160	0.5	2S_FA—1
		1	2S_FA—2
		1.5	2S_FA—3
		2	2S_FA—4
	190	0.5	3S_FA—1
		1	3S_FA—2
		1.5	3S_FA—3
		2	3S_FA—4

**Table 2:** Mechanical properties of composites with PET, rubber, HDPE, wood, and ash addition, before and after the water immersion.

T [°C]	Sample Code	*σ_t_* [N/mm^2^] Inițial	*σ_t_* [N/mm^2^] Immersion	R_c_ [N/mm^2^] Inițial	R_c_ [N/mm^2^] Immersion
150	1S_FA—1	1.66	1.68	63.62	58.67
	1S_FA—2	1.51	1.52	50.34	62.80
	1S_FA—3	1.52	1.69	55.08	50.91
	1S_FA—4	1.39	1.46	62.19	63.16
160	2S_FA—1	1.88	1.73	52.35	62.28
	2S_FA—2	1.68	1.84	66.60	58.01
	2S_FA—3	1.63	1.72	68.69	50.05
	2S_FA—4	1.58	1.68	66.55	53.12
190	3S_FA—1	2.09	2.07	57.42	56.52
	3S_FA—2	1.86	1.87	55.20	57.25
	3S_FA—3	1.89	1.87	61.16	51.67
	3S_FA—4	1.71	1.94	62.78	53.37

**Table 3:** Surface energies with dispersive and polar components for composites with fly ash unimmersed and water immersed.

Samples Code	Unimmersed					Water Immersed				
	Θ_water_ [^o^]	Θ_glycerol_ [^o^]	$\sigma_{SV}$ [mN/m]	$\sigma_{SV}^{d}$ [mN/m]	$\sigma_{SV}^{p}$ [mN/m]	Θ_water_ [^o^]	Θ_glycerol_ [^o^]	$\sigma_{SV}$ [mN/m]	$\sigma_{SV}^{d}$ [mN/m]	$\sigma_{SV}^{p}$ [mN/m]
1S	78.40	91.26	81.27	11.43	2.56	95.97	88.33	18.80	14.56	4.24
1S_FA—1	104.8	97.9	13.68	11.2	2.48	70.26	85.43	43.61	40.13	3.48
1S_FA—2	78.93	103	18.30	17.8	0.05	75.6	94.06	55.28	47.87	7.41
1S_FA—3	88.3	103.1	82.51	64.75	17.76	96.66	90.83	15.83	9.84	5.98
1S_FA—4	108.1	110.3	12.34	0	12.34	94.86	107.96	63.55	50.24	13.31
2S	102.70	94.65	16.63	14.45	2.18	88.84	73.47	32.60	28.39	4.20
2S_FA—1	108	99.3	16.06	15.14	0.92	82.3	96.16	82.86	68.66	14.21
2S_FA—2	91.2	95.6	12.88	12.27	0.61	82.33	86.43	35.16	0.04	35.14
2S_FA—3	107.3	101	11.72	9.22	2.51	93.5	96.73	23.73	23.72	0.01
2S_FA—4	108.1	102.2	10.92	8.3	2.62	96.06	105.4	42.85	37.39	5.46
3S	86.37	113.61	13.50	12.45	1.05	113.3	100.01	28.72	28.53	0.19
1S_FA—1	100.2	90.7	20.94	19.22	1.73	95.7	100.36	25.85	0.44	25.41
1S_FA—2	104.2	92	27.83	27.68	0.15	92.03	94.53	23.14	0.03	23.11
1S_FA—3	110.7	93.7	47.86	46.63	1.43	88.36	94.4	36.39	35.31	1.08
1S_FA—4	111.7	100.8	20.41	20.35	0.06	83.7	101.03	52.81	52.39	0.42

**Table 4:** FTIR bands of the composites with and with no FA added.

FTIR Bands	1S	1S_FA—2	Rubber	PET	HDPE	Wood
OH	3354	3327	-	-	-	3341
aliphatic C–H	2911	2907	2914		2914	2916
–CH=CH_2_	2845	2840	2847	-	2847	-
C=O	1714	1714	-	1713	-	1721
C=C	1610	1586 1531	1617	-	-	-
C=C rubber and HDPE. –CH_2_ of PET and CH of wood	1458	1428	1431	1407	1471	1421
C–C of rubber. CH_3_ of wood	-	1355	1372	-	-	1369
C–O–C from PET and wood	1245	1215	-	1240	-	1245
C–O–C wood	1093	1069				
Si-O stretching vibration (FA)		1115				
C–O–C wood	1016	1020		1016		1025
aromatic nuclei in PET	837	874 813	-	872	-	-
C-H	717	717		723	717	-
FA		537				

**Table 5:** Representative band values in the IR spectra of water-immersed fly ash composites.

Specific Groups	150 °C	150 °C. 1% Fly Ash	150 °C. 1% Fly Ash Immersed	Rubber	PET	HDPE	Wood
OH	3354	3327	3284	-	-	-	3341
C-H aliphatic	2911	2907	2908	2914		2914	2916
–CH=CH_2_	2845	2840	2842	2847	-	2847	-
C=O	1714	1714	1722	-	1713	-	1721
C=C	1610	1586 1531	1584 1531	1617	-	-	-
C=C rubber and HDPE. –CH_2_ in PET și CH in wood	1458	1428	1430	1431	1407	1471	1421
C-C in rubber. CH_3_ in wood	-	1355	1369	1372	-	-	1369
C-O-C in PET and wood	1245	1215	1219	-	1240	-	1245
Si-O stretching vibration (FA)		1115	1118				
C-O-C in wood	1093	1069					
C-O-C in wood	1016	1020	1019		1016		1025
aromatic nuclei in PET	837	874 813	815	-	872	-	-
C-H	717	717	719		723	717	-
metal oxides		537	552				
